# Establishment of a Human Immunocompetent 3D Tissue Model to Enable the Long-Term Examination of Biofilm–Tissue Interactions

**DOI:** 10.3390/bioengineering11020187

**Published:** 2024-02-15

**Authors:** Rasika Murkar, Charlotte von Heckel, Heike Walles, Theresia Barbara Moch, Christoph Arens, Nikolaos Davaris, André Weber, Werner Zuschratter, Sönke Baumann, Jörg Reinhardt, Sascha Kopp

**Affiliations:** 1 Core Facility Tissue Engineering, Otto-von-Guericke University Magdeburg, Universitätsplatz 2, 39106 Magdeburg, Germany; 2 Department of Otorhinolaryngology, Head and Neck Surgery, University Clinic Giessen, 35392 Giessen, Germany; christoph.arens@hno.med.uni-giessen.de (C.A.);; 3 Photonscore GmbH, Brenneckestr. 20, 39118 Magdeburg, Germany; 4 Leibniz Institute for Neurobiology, 39118 Magdeburg, Germany; 5 Omicron-Laserage^®^ Laserprodukte GmbH, Raiffeisenstr. 5e, 63110 Rodgau, Germany; 6 MedFact Engineering GmbH, Hammerstrasse 3, 79540 Lörrach, Germany

**Keywords:** human 3D tissue model, biofilm, immunocompetent, macrophages, tissue engineering, primary cells

## Abstract

Different studies suggest an impact of biofilms on carcinogenic lesion formation in varying human tissues. However, the mechanisms of cancer formation are difficult to examine in vivo as well as in vitro. Cell culture approaches, in most cases, are unable to keep a bacterial steady state without any overgrowth. In our approach, we aimed to develop an immunocompetent 3D tissue model which can mitigate bacterial outgrowth. We established a three-dimensional (3D) co-culture of human primary fibroblasts with pre-differentiated THP-1-derived macrophages on an SIS-muc scaffold which was derived by decellularisation of a porcine intestine. After establishment, we exposed the tissue models to define the biofilms of the *Pseudomonas* spec. and *Staphylococcus* spec. cultivated on implant mesh material. After 3 days of incubation, the cell culture medium in models with M0 and M2 pre-differentiated macrophages presented a noticeable turbidity, while models with M1 macrophages presented no noticeable bacterial growth. These results were validated by optical density measurements and a streak test. Immunohistology and immunofluorescent staining of the tissue presented a positive impact of the M1 macrophages on the structural integrity of the tissue model. Furthermore, multiplex ELISA highlighted the increased release of inflammatory cytokines for all the three model types, suggesting the immunocompetence of the developed model. Overall, in this proof-of-principle study, we were able to mitigate bacterial overgrowth and prepared a first step for the development of more complex 3D tissue models to understand the impact of biofilms on carcinogenic lesion formation.

## 1. Introduction

Bacterial composition in biofilms is thought to have an influence on the differentiation of malignant cells and contribute to tumour initiation, promotion, and progression. Results of investigations on cancer formation suggest a correlation between biofilms and several cancer types such as colorectal, intestine, or oral cancer [1].

One of the main mechanisms seemed to be that of the changes in cell metabolism and DNA due to chronic inflammation, which are triggered by biofilms [2]. Another mechanism might be that of the synthesis of carcinogenic substances by bacteria [3]. As an example, *Pseudomonas aeruginosa* is considered to play a role in the development of oral cancer as it synthesises nitric oxide, which is thought to be involved in cancer progression [4]. Furthermore, it should be noted that *P. aeruginosa* is a Gram-negative bacterium that is capable of forming biofilms. In addition, there has been more research on the biofilm formation of staphylococci, such as *Staphylococcus epidermidis*, which are the main pathogens associated with biofilm infections on implanted medical devices [5]. Biofilms are accumulations of microorganisms, particularly bacteria, embedded in a self-produced matrix of proteins, polysaccharides, and eDNA. They can form on biological surfaces, such as skin, as well as on products like medical devices and are therefore in continuous direct contact with human tissues [1,6].

To enable investigations into the tissue remodelling of bacteria in human tissues, immunocompetent 3D tissue models come in handy. Among others, such models include immunocompetent organ-on-a-chip models, organoids, animal models, and co-cultures of immune cells. However, these models bring drawbacks with them such as by not fully reproducing human physiology (especially in animal models). Organ-on-a-chip models, though, can better mimic the physiological functions of tissues and organs using microfluidic technologies, but the complex structure of immune tissues presents a challenge [7]. Organoids have a limited lifespan as well as being very expensive and time-consuming to culture [8]. Three-dimensional in vitro tissue models are crucial for different investigative studies, and there is a wide spectrum of methodologies to produce such tissues. Apart from synthetic approaches like lab-on-chip and 3D bioprinting technologies, other active cell patterning methods, like that of acoustic-assisted bioassembly, are researched (surface acoustic waves (SAWs)) to produce tissue-like cell constructs [9,10]. Secondly, a physiological extracellular matrix (ECM) is missing in most models. The ECM, however, enables tissue and cell polarization and is needed for cell–cell, cell–ECM communication, and cell migration [11]. Biologically derived scaffolds, such as SIS-muc, provide more of a physiological resemblance towards the required ECM and native cellular behaviour [12]. Such 3D models could also prove to be a better choice over 2D approaches given the benefits of 3D orientation for cells, cellular differentiation, improved barrier functions, among others (Table 1) [13].

Fibroblasts and macrophages are universal cell types, which are found in nearly every tissue. They are key cell types for tissue homeostasis and the first to react to inflammation [14]. While fibroblasts’ major task is the secretion and remodelling of the extracellular matrix, during an inflammatory event, they pave the way for tissue organisation, cell polarization, cell migration, and immune cell attraction through chemokine secretion [15].

Macrophages, on the other hand, are a key type of innate immune cells within the immune system. Their main function is that of phagocytosis. These cells can polarise into two main functional phenotypes, which are called pro-inflammatory M1 macrophages and anti-inflammatory M2 macrophages, depending on the surrounding microenvironment [16]. M1 macrophages are responsible for efficiently killing pathogens, while M2 macrophages are those that repair and heal tissue [17]. Both macrophage phenotypes polarise from non-polarised M0 macrophages [18]. It is believed that the macrophage population is heterogeneous and dynamic because of the rapid and reversible polarisation process [16].

In this study, we focus on the establishment of an immunocompetent human 3D tissue model based on a combination of human fibroblasts and pre-differentiated macrophages on a decellularized SIS-muc scaffold [19], which should allow for a co-cultivation with a representative bacterial biofilm formed from *Pseudomonas stutzeri* and *Staphylococcus simulans* for long-term observations. The establishment of such an immunocompetent 3D tissue model can be beneficial for fundamental as well as applied sciences while enabling a physiologically relevant environment. In addition to researching communications between human tissues and microorganisms, it can also be used to investigate the interface between biofilms and overgrown implants and tissue, resulting in being a valuable tool for medical device development, sterilisation processes, and validations.

**Table 1:** Comparison between 2D model systems and 3D model systems with regard to the impact of bacterial exposure [20,21,22,23].

3D Model System	2D Model System
Improved proliferation and differentiation	Loss of cellular phenotype
Bacteria can surpass into deeper tissues	Change in morphology and functionality
Interaction with fibroblasts	Loss of cell signalling
Cell–cell and cell–ECM interactions	Standardizable

## 2. Materials and Methods

### 2.1. Cell Culture

#### 2.1.1. THP-1 Differentiation

THP-1 cells were initially derived from the European Collection of Authenticated Cell Cultures and kindly donated by the thoracic surgery department of the university clinic in Magdeburg. Cells in a density of 1 × 10^5^ were seeded in each well of a 12-well plate with coverslips and incubated for one hour with a THP-1 medium consisting of RPMI-1640 with 2 mM of L-glutamine (Gibco, Darmstadt, Germany, 21875034) + 10% FCS (Bio&Sell, Feucht bei Nürnberg, Germany, FBS.S0615) + 1% PenStrep (Sigma-Aldrich, Taufkirchen, Germany, P0781). Phorbol-12-myristat-13-acetat (PMA, Sigma-Aldrich, Taufkirchen, Germany, P1585) with a working concentration of 50 ng/mL was added and incubated for 24 h. After 24 h, the PMA-containing medium was removed and the cells were washed with the THP-1 medium three times. Then, the THP-1 medium was added and incubated for 1 h. For differentiation in the M0, M1, and M2 macrophages, different mediums were used as follows: M0 (THP-1 medium), M1 (THP-1 medium + IFN-y (50 ng/mL), Sigma-Aldrich, Taufkirchen, Germany, SRP3058) and M2 (THP-1 medium + IL4 (Sigma-Aldrich, Taufkirchen, Germany, SRP4137) + IL13 (Sigma-Aldrich, Taufkirchen, Germany, SRP3274) (each 25 ng/mL)). Differentiation in the cells was achieved after 48 h [24].

#### 2.1.2. Primary Fibroblasts

Primary cell isolation from fascia biopsies was performed under the approval of the Local Ethics Committee of the University of Wuerzburg (182/10) and with the informed consent of the patients. Fibroblasts were cultivated in DMEM high glucose (Sigma-Aldrich, Taufkirchen, Germany, D5796) supplemented with 10% FCS (Bio&Sell, Feucht bei Nürnberg, Germany, FBS.S0615) under standard conditions (37 °C, 5% CO_2_). 

#### 2.1.3. SIS-muc

A collagen-based matrix derived from the pig intestine was used (SIS-muc, Fraunhofer Institute Würzburg, Germany). This was fixed in cell crowns as previously described [19].

### 2.2. Bacteria Culture

For bacterial enrichment, we used representatives of *Pseudomonas* spec. and *Staphylococcus* spec., which are known to form biofilms [25,26]. Visually equal colonies of *Staphylococcus simulans* (ATCC 27851) and *Pseudomonas stutzeri* (ATCC 17588) were taken from each strain with an inoculation loop and mixed in centrifuge tubes with CASO Bouillon (TSB) Ph. Eur. (Chemsolute, Th Geyer, Renningen, Germany, 9721.0500). Uniform pieces of the Optilene^®^ Mesh Elastic (B.Braun, Melsungen, Germany) were taken with a 5 mm punch (Mediware, 03805980 servoprax GmbH, Wesel, Germany), and one mesh section was placed into separate centrifuge tubes. After 48 h of incubation at 30 °C, the centrifuge tubes were vortexed, and the meshes were then placed on agar plates (Blutagar (Basis) (Chemsolute, Th Geyer, Renningen, Germany, 9850.0500)).

### 2.3. Establishment of a 3D Model

**Figure 1:**
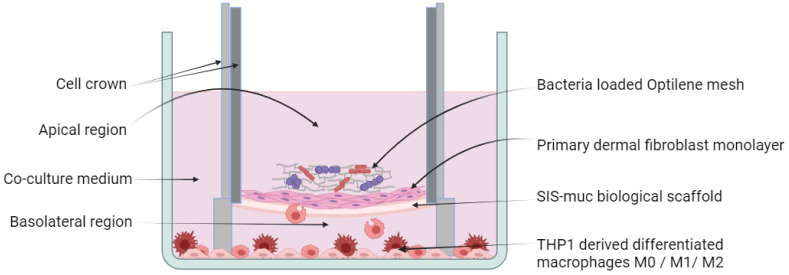
Schematic overview of the 3D immune-competent tissue model system. Three-dimensional tissue models are developed using biologically derived scaffold SIS-muc with primary human fibroblasts having strong cell–cell and cell–ECM contacts. Such a tissue model is held upright using a metal cell crown, as shown in the figure with the fibroblastic monolayer facing the apical side. Co-cultures are setup with M0/ M1/ M2 macrophages. Such a tissue model is exposed to distinctive bacterial colonies grown on an Optilene mesh. Image drawn in BioRender.

Primary fibroblasts with a seeding density of 5 × 10^5^ cells/cm^2^ were cultured on each SIS-muc model on the apical side. After 24 h, the cell crowns were placed in the well plate with the differentiated macrophages and cultured for 11 days. The medium exchange was undertaken three times a week and consisted of a THP-1 medium and a fibroblast medium in a 50:50 ratio. On the 11th day of the co-culture, supernatant samples from each model were collected and marked as “Day 0”. Furthermore, two parallel assays were setup, where one consists of co-cultured models with bacteria-loaded Optilene ^®^ meshes (see Figure 1) and the meshes without bacteria were used for the other set of models. Such a co-culture setup was incubated for another 3 days. At the end of the 3rd day (day 14), supernatant samples were collected from each of the co-cultured models labelled as “Day 3 (with bacteria)” and “Day 3 (without bacteria)” and were stored for further analysis. In addition, 3D models were collected and fixed for further analysis.

### 2.4. Qualitative Analysis

Histological, immunohistochemical, and immunofluorescent analyses were performed. The tissue models were embedded in Tissue-Tek O.C.T Compound (Sakura, Finetek USA, Torrance Canada 4583), stored at −80 °C, and sectioned at a 10 μm thickness. The samples were stained with hematoxylin and eosin according to standardized protocols.

#### 2.4.1. Immunohistochemical Analysis

Analysis was performed using the Super Vision 2 HRP Kit (DCS, Hamburg, Germany, PD000KIT) according to manufacturer protocols.

The slides were incubated with blocking buffer (PBS- + 0.5% BSA) at room temperature for one hour. Thereafter, incubation of primary antibodies vimentin and Ki67 was performed according to Table 2. The staining protocol had to be optimized for primary antibodies CD68, CD80, and CD163 since the blocking was carried out with PBS- + 3% BSA for six hours at 4 °C to reduce background staining. Mouse IgG serum (Sigma-Aldrich, Taufkirchen, Germany, I8765) and rabbit IgG serum (Sigma-Aldrich, Taufkirchen, Germany, I8140) were used as negative controls. 

#### 2.4.2. Immunofluorescence Staining

Staining was performed for macrophage characterization using the respective CD markers for M0, M1, and M2 macrophages, as shown in Table 2, as well as for cellular tissue characterization on the fixed in vitro tissue models. 

Tissue sections were stained using the mixture of a fibroblast-specific primary antibody with the condition-specific (M0/M1/M2) primary antibodies diluted according to the given dilution in Table 2. Briefly, after blocking using 5% FCS + 1% BSA in PBS- for 4 h, these primary antibodies (for incubation time, refer to Table 2) were counterstained using host-specific fluorescently labelled secondary antibodies. Finally, nuclei were stained blue using DAPI.

Similarly, THP-1 differentiated macrophage characterization was also performed on the cells differentiated in a 12-well plate separately using standardized protocols [24]. Briefly, the cells were gently washed with PBS- and fixed in Paraformaldehyde (PanReac AppliChem, Darmstadt, Germany, 256462) for 10 min, which was followed by permeabilization (0.5% Tween20 in PBS-) and blocking steps with intermittent washing with PBS-. Following 1 h of blocking at room temperature, cells were coated with the respective primary antibodies (see Table 2) and counterstained with host-specific fluorescently labelled secondary antibodies and DAPI nuclei staining. Fluorescence imaging was performed using a ZEISS Axio Observer fluorescence microscope (ZEISS, Oberkochen, Germany).

### 2.5. Quantitative Analysis

#### 2.5.1. OD Measurement

The depleted cell culture medium obtained from the wells of the tissue models was stored, and an optical density (OD) measurement at 580 nm was carried out to determine the turbidity levels of the medium. The turbidity/the optical density value provides information about the bacterial contamination of the medium and was therefore used to judge the bacterial content. Therefore, 1 mL of each medium was pipetted into cuvettes and analysed in a photometer (Grant-bio DEN-600, Riga, Latvia). A cuvette with 1 mL of fresh fibroblast medium was used as the blank. 

#### 2.5.2. Agar Plates

The medium was spread on agar plates (Blutagar (Basis) (Chemsolute, Th Geyer, Renningen, Germany, 9850.0500)) with sterile 10 μL inoculation loops using the streak plate technique. The plates were incubated for 24 h.

#### 2.5.3. ELISA

Multiplex ELISA based on the bead assay technique was utilised to quantify the expression of 13 different cytokines known to play a vital role in immune responses. The protocol was adapted as described by the manufacturer to be used for the “Essential Immune response panel” (Biolegend, Amsterdam, The Netherlands, 740930). This entire panel consists of 13 key target cytokines for the detection of IL-2, IL-4, IFN-γ, TNF-α, free active TGF-β1, IL-10, IL12p70, IL17A, IL-1β, CXCL8 (IL-8), CXCL10 (IP-10), CCL2 (MCP-1), and IL-6. The assay was performed using a V-bottom 96-well plate. Supernatants collected and labelled as “Day 0”, “Day 3 with bacteria”, and “Day 3 without bacteria” from the three conditions of M0, M1, and M2 were used in an undiluted form for cytokine analysis. 

#### 2.5.4. Image Processing and Quantification of IHC Images

Image processing was carried out using the Image Processing Toolbox™ [27] with MATLAB R2022a (The MathWorks, Inc.). Representative images were processed individually. The raw RGB images were converted into grayscale images, and the contrast of the grayscale images was enhanced linearly. Segmentation of the area corresponding to the tissue was carried out on the enhanced grayscale image by setting a manual threshold. The segmented images were visually inspected to ensure a successful segmentation process. Identification of the area within the tissue that was positively stained was carried out using a fixed threshold on the segmented, non-enhanced grayscale image (Figures S1–S3).

The percentage of tissue stained positively was then defined as follows:

$$\%\;of\;IHC\;positive\;tissue=\frac{IHC\;positive\;area\;within\;the\;tissue\;(Pixels)}{Area\;of\;the\;tissue\;(Pixels)\;}\times 100$$

### 2.6. Statistics

The statistical evaluation was performed using the software OriginPro 2023b. 

The samples with and without bacteria and the differences between “Day 0” and “Day 3” were analysed with the non-parametric Mann–Whitney U test. *p*-values < 0.05 were considered to be significant, with *p* < 0.01 being highly significant.

Statistical evaluation for ELISA was undertaken as follows: Samples collected from two experimental runs were analysed in Multiplex ELISA in triplicate forms (sample number 6). Statistical analysis was performed using standard MS Excel software (Microsoft, Office 2016). The data collected did not follow normal distribution, and the sample populations were assumed to be independent samples. Hence, a non-parametric statistical test for three sample populations known as the Kruskal–Wallis ANOVA test was performed to compare the three populations namely on Day 0, Day 3 without bacteria, and Day 3 with bacteria for each and every measured cytokine. *p* values < 0.05 were assumed to be showing a significant difference in their expression between Day 0, Day 3 without bacteria, and Day 3 with bacteria for the respective cytokine in the given condition of either M0/M1/M2. Furthermore, the Mann–Whitney Wilcoxon rank sum non-parametric test was performed to determine, if the expression of a particular cytokine is significantly higher or lower between Day 0, Day 3 without bacteria, and Day 3 with bacteria. Again, *p* values < 0.05 were assumed to be showing a significant increase or decrease.

## 3. Results

### 3.1. Differentiation of THP-1 Monocyte-Like Cells into Macrophages

The first step of the establishment of the model was to differentiate the THP-1 monocyte-like cells into M0, M1, and M2 macrophages. To ensure an efficient and controlled differentiation of THP-1 monocytes into the respective phenotypes, immunofluorescence images were taken. To discriminate the phenotypes, specific surface markers were stained, as listed in Table 2. The respective serum was used in exchange with the primary antibody, to ensure that no unspecific binding of the secondary antibodies appeared. Counterstaining for nuclei was carried out with DAPI.

**Figure 2:**
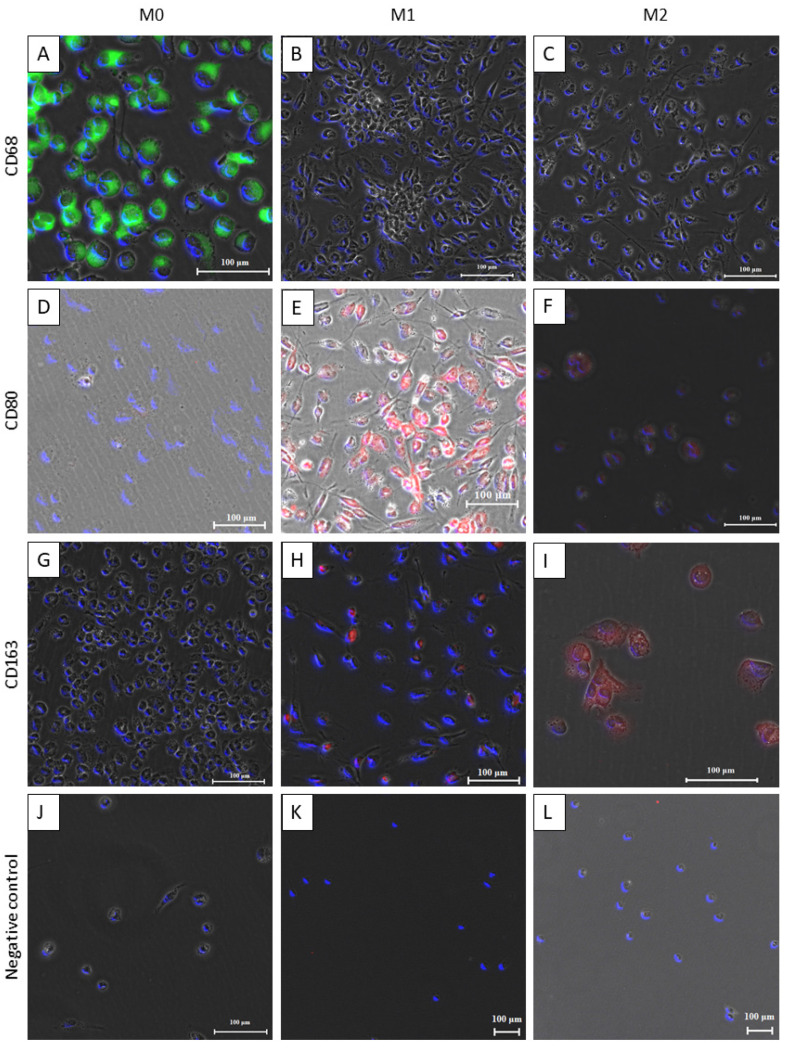
M0, M1, and M2 functional characterization using cell surface-specific markers. The THP-1 differentiated macrophage phenotypes, which are M0, M1, and M2, are functionally characterized using cell-specific markers for M0 with CD68, M1 with CD80, and M2 with CD163. DAPI is used as a blue nuclear counterstain. Serum was used as a negative control. Schemes follow the same formatting. (**A**–**J**) 20× magnification; (**K**,**L**) 10× magnification. (For each differentiation status, three images from random positions were taken).

The THP-1-derived M0 macrophages were stained positive for CD68 and labelled with the FITC secondary antibody (Figure 2A), while CD80 and CD163, representing markers for M1 and M2 macrophages, respectively, appeared to be negatively stained (Figure 2D,G). The M1 differentiated macrophages were stained positive for the CD80 cellular marker (Figure 2E). On the contrary, certain M1 macrophages were also found to be positive for the CD163 M2 cell marker (Figure 2H). The M2 differentiated macrophages were stained positive for CD163 marker (Figure 2I). In addition, certain cells were also stained positive for CD80, representing a small subset of M1 cells within the M2 population (Figure 2F). The negative controls showed no staining for any secondary antibody (Figure 2J–L). In addition, the M1 macrophages appear more spindle-shaped and elongated when compared with the shape of the M0 macrophages, which appeared more circular. Comparing Figure 2A–C, the M0 differentiated macrophages were visually smaller in size than the M2 macrophages were.

### 3.2. Immunocompetent Tissue Model (Bacteria)

Bacterial contamination and overgrowth in cell cultures is usually accompanied by a colour change and a milky appearance of the cell culture medium. To determine the degree of contamination for the 3D tissue model after three days of incubation with defined biofilms, a visual examination, optical density measurement of the medium, and streak test have been performed.

**Figure 3:**
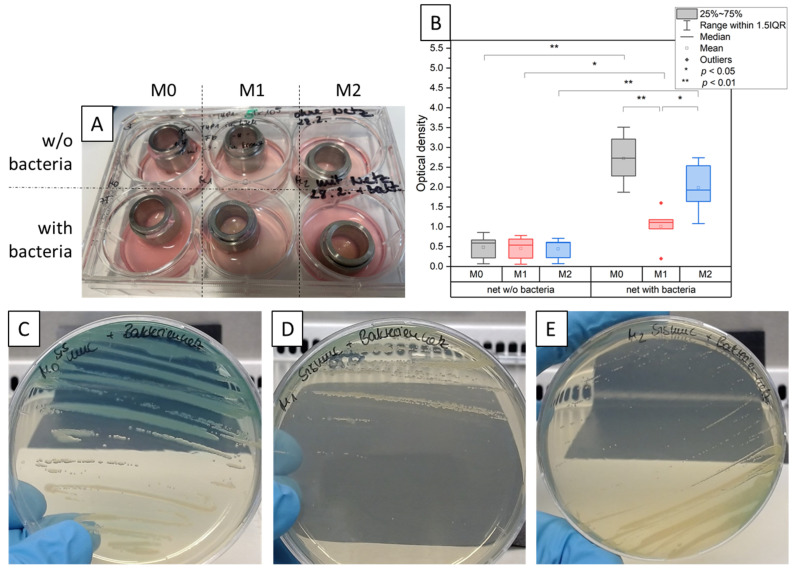
Bacteria test. (**A**) Tissue models with different macrophage differentiation stages with and without bacterial exposure. Optically different turbidity levels of the medium are presented. (**B**) Optical density (OD) measurements of the depleted cell culture medium of the different models for judgement of bacterial content. The models with M0 macrophages have the highest OD value, and those with M1 have the lowest, with *n* = 5, * *p* < 0.05, ** *p* < 0.01. (**C**–**E**) Spreading of the cell culture medium on agar plates. (**C**) The plate with the highest growth of bacteria is the M0 plate. (**E**) For M2, less colonies are visible, and (**D**) bacterial growth was the lowest for M1.

Figure 3A shows the turbidity of the cell culture medium for the different tissue models with and without bacterial exposure. It is visible that the medium with M0 macrophages has the highest turbidity. The medium with M2 macrophages is less turbid, and the medium with M1 does not show any major optical differences to the medium without bacteria. The optical density (OD) measurements of the models without bacteria show similar values of around 0.5 (Figure 3B). However, the models with bacteria show a significant difference between M0, M1, and M2 to be visible. For M0 and M2, a high significance is shown between the values without bacteria and with bacteria where M0 has the highest OD value with a mean of 2.7. M1 is slightly increased compared with the medium without bacteria with a mean value of 1.0, and it is therefore only barely significant. When spreading the medium on agar plates, a difference in bacterial growth is also visible. The strongest bacterial growth can be seen on the plate with the M0 medium (Figure 3C). Figure 3D (M1 model) shows the least growth, and Figure 3E (M2 model) presents moderate growth.

To determine the impact of the bacterial biofilms on the tissue model, the overview HE as well as vimentin immunohistochemical staining were performed.

**Figure 4:**
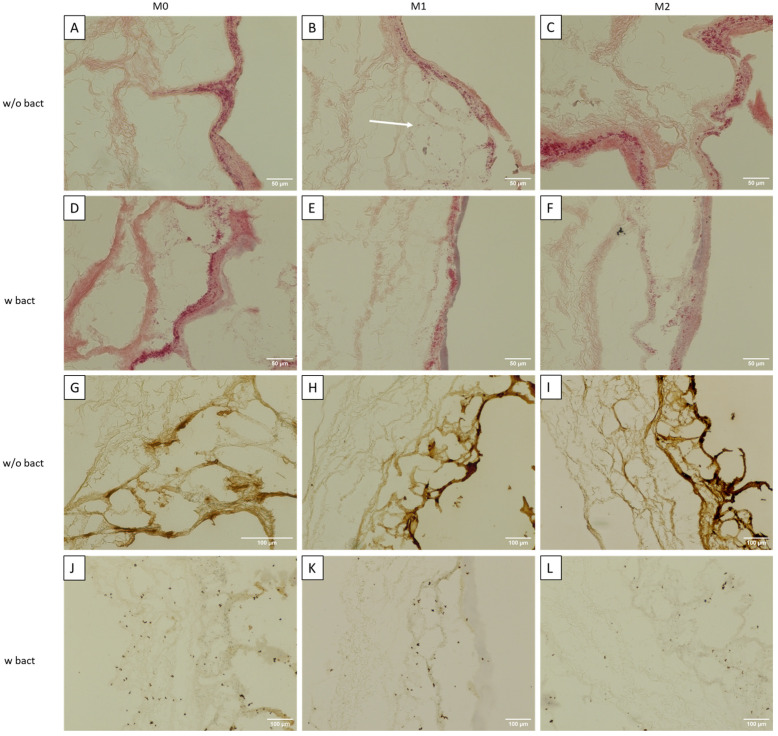
HE staining of tissue models with M0, M1, and M2 macrophages without infection (**A**–**C**) and with bacteria (**D**–**F**). Immunohistochemical analysis with the antibody vimentin for healthy tissue (**G**–**I**) and after infection (**J**–**L**). (**A**–**G**) 40× magnification; (**H**–**L**) 20× magnification. *n* = 3–5 images per model.

Figure 4A presents the tissue model with M0 macrophages without a bacterial load. A dense tissue is visible. The tissue with M1 macrophages (Figure 4B) is clearly more loosened. Cellular structures are not only present on the matrix but have also migrated into the SIS-muc scaffold. The tissue model with M2 macrophages (Figure 4C) is comparable to the M0 tissue model as it also shows very dense structures here. With bacteria, on the other hand, tissue models with M0 and M1 macrophages (Figure 4D,E) look rather similar. In both tissue models, the staining is not visible anymore. In M1, the staining is visible on the surface, but cellular structures are still apparent underneath. For the M0 model, the surface is also lysed, and the matrix has dense structure. In the M2 model (Figure 4F), lysis is also visible, and there are barely any cellular structures left. For the Vimentin staining, many active fibroblasts are stained in the tissue models without bacteria (Figure 4G–I), which are shown through dense brown structures. Post-processing and quantification of the ratio between the tissue area stained in brown and the total tissue area presented the 9.87% positively stained region in the M0 models, the 17.73% one in the M1 models, and the 26.53% one in the M2 models. In the tissue models with the bacterial meshes, there are not as many brown-stained locations visible. In the models M0 and M1 (Figure 4J,K), certain cells are still present representing the 1.21% and 0.92% positively stained regions, which is noticeably less in the model with M2 with a 0.56% positively stained region (Figure 4L).

**Figure 5:**
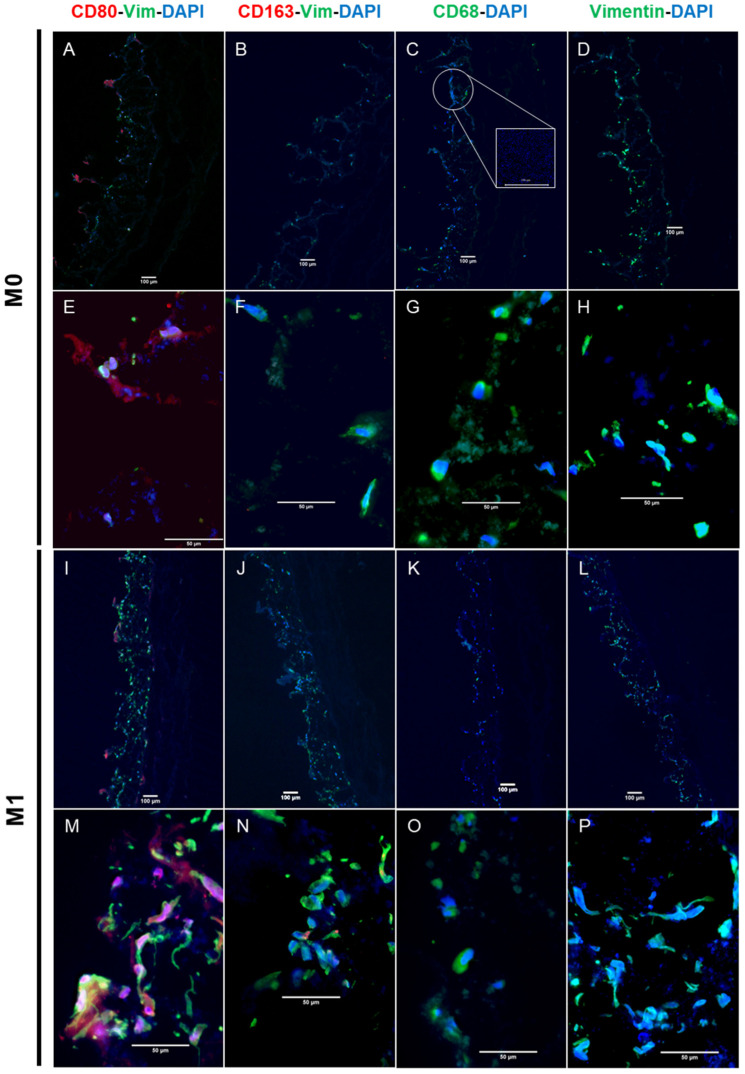

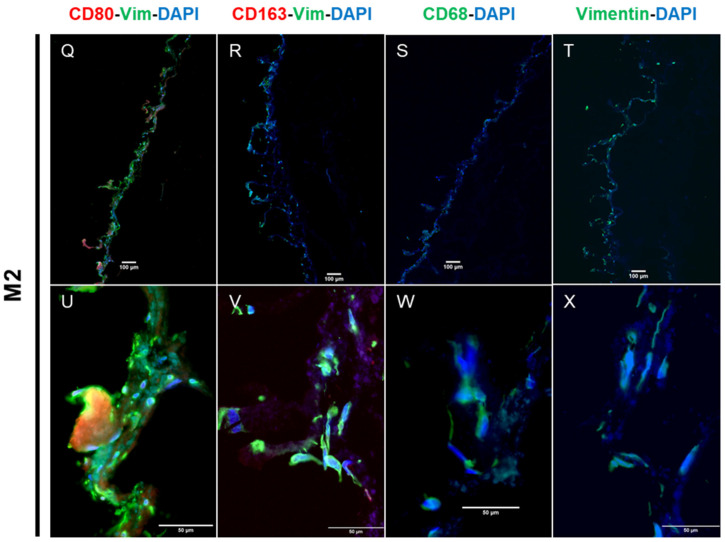
Influence of the THP-1 cell line-derived macrophages in three functionally pre-differentiated states, namely M0 (figures (**A**–**H**)), M1 (figures (**I**–**P**)), and M2 (figures (**Q**–**X**)) when in co-culture with 3D tissue with a bacterial biofilm. All figures show the nucleus and other DNA materials counterstained in blue using DAPI. The overview 5× magnification (**A**–**D**; **I**–**L**; **Q**–**T**) and corresponding 40× magnification detailed images (**E**–**H**; **M**–**P**; **U**–**X**) (*n* = 3–4 images). Figures in the first column ((**A**) and (**E**); (**I**) and (**M**); (**Q**) and (**U**)) represent the sections stained for the M1 marker CD80 with red fluorescently labelled secondary antibodies along with vimentin (green) and DAPI (blue). Figures in the second column ((**B**) and (**F**); (**J**) and (**N**); (**R**) and (**V**)) represent the sections stained with the M2 marker CD163 with red fluorescently labelled secondary antibodies along with vimentin (green) and DAPI (blue). Figures ((**C**) and (**G**); (**K**) and (**O**); (**S**) and (**W**)) represent the sections stained for the M0 marker CD68 with green fluorescently labelled secondary antibodiws and DAPI (blue). Additionally, figures ((**D**) and (**H**); (**L**) and (**P**); (**T**) and (**X**)) represent the sections stained for vimentin (green) along with DAPI (blue).

To show that the pre-differentiated macrophages migrate into the tissue model, immunofluorescence staining was performed. Figure 5 shows the immunofluorescence staining of the tissue sections where fibroblasts are stained by antibodies against vimentin in green. M1 and M2 macrophages are stained using their respective markers CD80 and CD163 by red fluorescently labelled secondary antibodies, and M0 macrophages are stained with a CD68 marker using green fluorescently labelled secondary antibodies, which can be seen close to the cell nuclei. Nuclei are counterstained by DAPI in blue.

On tissues in co-culture with M0 macrophages, the bacterial biofilm was observed in the form of a blue smear, as shown in Figure 5C. Meanwhile, certain M0 macrophages stained with CD68 marker migrated into the tissue (Figure 5G). Additionally, positively stained CD80 revealed the presence of certain M1 macrophages, as shown in Figure 5A,E, while a low number of vimentin-positive (green) stained fibroblasts can be pinpointed in Figure 5D,H.

On the contrary, tissues in co-culture with M1 macrophages showed a comparatively higher number of vimentin-positive stained fibroblasts (Figure 5N,P). In addition to the presence of CD80 positively stained M1 macrophages (Figure 5M), certain M0 macrophages were also seen to be stained positive for CD68 (green) (Figure 5O). 

Furthermore, for the tissues in the co-culture with M2 macrophages, little to no macrophages were found to be stained positive for the M2 macrophage marker CD163 (red), while the CD80 (red) positively stained M1 macrophages were observed as shown in Figure 5U along with certain M0 macrophages that had stained positively for CD68 (green) in Figure 5W. Moreover, most of the vimentin-stained fibroblasts showed a green-smeared appearance and a disintegrated cytoskeleton structure (Figure 5U,X), while the appearance of the biofilm smear was evidently seen to be similar to that with tissue sections in a co-culture with M0 macrophages.

### 3.3. Cytokine Secretion

**Figure 6:**
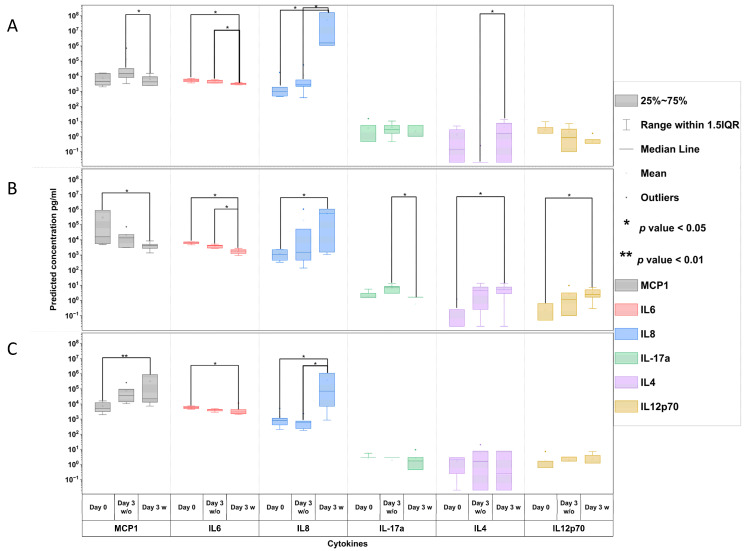
Concentrations of six selected cytokines, namely MCP1, IL6, IL8, IL-17a, IL4, and IL12p70, for the co-cultured models with THP-1-derived (**A**) M0 macrophages, (**B**) M1 macrophages, and (**C**) M2 macrophages. Concentrations of the abovementioned cytokines at “Day 0”, which is from the control models without a bacterial net, and at “Day 3 w/o” for models at day 3 without a bacterial net are compared against the concentration at “Day 3 w” for models at day 3 with a bacteria-infested net, which is depicted using the logarithmic scale on the Y-axis. (Experimental repetition = 3, and probes from two experiments were used in triplicate for the ELISA analysis. Due to the lower number of samples, statistics were performed using the Kruskal–Wallis ANOVA non-parametric test comparing all three variants for each cytokine and the Mann–Whitney test comparing concentrations at day 3 with control groups without bacteria, with * *p* < 0.05 and ** *p* < 0.001).

A bead-based assay was used to detect and quantify a panel of cytokines and chemokines commonly seen in an immune response. The use of the multiplex ELISA technique based on antibody-immobilized as well as size- and fluorescence-encoded beads enables the simultaneous detection of over 13 different cytokines and chemokines in a sample in one single run. The Legend-Plex essential immune response panel comprises the detection of IL-2, IL-4, IFN-γ, TNF-α, free active TGF-β1, IL-10, IL12p70, IL17A, IL-1β, CXCL8 (IL-8), CXCL10 (IP-10), CCL2 (MCP-1), and IL-6. These cytokines are seen to be involved in the immune response (inflammatory or anti-inflammatory) during inflammation as well as a foreign body reaction [28]. Figure 6 shows the cytokines and chemokines, which were measured to be significantly changes in the experimental conditions.

The concentration of MCP-1 in M0 macrophage models increased after 3 days of cultivation without a bacterial load. However, the M0 macrophage models that were in contact with the biofilms presented a significantly lower concentration of MCP-1 compared with the non-biofilm models, but then these were equally high on day 0 (Figure 6A). MCP-1 concentrations for M1 macrophage models presented a lower level after 3 days of cultivation without bacteria and a significant decrease after cultivation with bacteria (Figure 6B). In contrast, in the presence of M2 macrophages, MCP1 expression showed a significant (100×) increase at day 3 in the models incubated with bacterial film when compared with that of the models at day 0 and day 3 without bacteria.

IL6 expression decreased significantly at day 3 when exposed to bacteria in the models kept in a co-culture with M0 macrophages (Figure 6A) as well as in both the M1 macrophages (Figure 6B) and the co-culture with M2 macrophages (Figure 6C). 

IL8 expression significantly increased on day 3 in the models incubated with bacterial film when compared with that observed for day 0 and day 3 in the models without bacterial exposure in a co-culture with M0 macrophages (Figure 6A). Similarly, in the case of the co-culture with M1 macrophages (Figure 6B), IL8 expression increased significantly at day 3 in the models exposed to bacterial film but was not as high as that seen for models in a co-culture with M0, as shown in Figure 6A. Moreover, the M2 macrophage models (Figure 6C) present the IL8 expression to have significantly increased at day 3 in the models with bacterial film when compared with the models at day 0 and day 3 without the bacterial film. However, this was not as high as that which was seen in the models that were kept in a co-culture with M0 macrophages, as shown in Figure 6A.

Meanwhile, in the M0 macrophage models (Figure 6A), IL4 expression decreased and was found to be unexpressed at day 3 in the same models without bacteria. Bacterial exposure for 3 days significantly increased the expression of IL6. In the co-cultured M1 macrophage models (Figure 6B), IL4 expression followed an increasing trend when without bacteria and demonstrated a significant increase at day 3 in the models that were exposed to the bacterial film when compared with the models at the experimental start without bacteria. Contrarily, in the models in a co-culture with M2 macrophages (Figure 6C), IL4 expression was seen to be unvaried.

While IL17a and IL12p70 expression remained unchanged even with the inclusion of bacterial biofilms in the models in a co-culture with M0 macrophages (Figure 6A), IL17a presented significant diminishment at day 3 in the models incubated with the bacterial film when in a co-culture with M1 macrophages (Figure 6B). Meanwhile, a low amount of IL17a was also expressed at day 3 in the models with bacteria when in a co-culture with M2 macrophages (Figure 6C), which was absent at day 0 and day 3 in models without the bacterial net.

IL12p70 expression increased significantly at day 3 in the models incubated with the bacterial film in a co-culture with M1 macrophages (Figure 6B). Contrarily, in the case of the co-culture with M0 (Figure 6A) and M2 (Figure 6C) macrophages, IL12p70 remained unvaried.

## 4. Discussion

An immunocompetent human 3D tissue model can be a valuable tool to unravel the strongly discussed impact of biofilms on the formation of cancer lesions [29]. Biofilms produced by a wide spectrum of bacteria are a product of their survival strategy. Biofilms are clusters of bacteria attached to surfaces, which are enveloped in a matrix composed of proteins, polysaccharides, and eDNA. Bacteria within biofilms employ various strategies to elude the immune system, adapt to limited oxygen and nutrients, and develop resistance to antimicrobial treatments. These infections trigger immune responses but are seldom resolved, leading to persistent, slow-developing diseases with inconsistent responses to therapies [6]. Many studies are now trying to prove links between the role of biofilms in the pathogenesis of diseases and cancerous lesion formations in different bodily systems including the cardiovascular, digestive, and more vital systems [1]. In vitro tissue engineering contributes various advanced methods to models similar to the in vivo immune response generated by bacterial biofilms by applying techniques ranging from 2D cultures of primary human cells, organoid cultures, and the most recently developed one of organ-on-chip technology [29]. Further research aims to develop an in vitro model that offers a more physiological and immunocompetent microenvironment for the efficient investigation of microbial interactions, immune responses, pathogenicity mechanisms, and cellular dysfunction due to changes in microbial compositions. One such 3D intestinal immunocompetent model was developed using organ-on-chip technology and was compared with those developed using organoid and 2D techniques [29]. Among certain stated limitations, the model utilised the epithelial colorectal cancer cell line Caco-2, and while colonization from *L. rhamnosus* did not induce inflammatory responses by release of pro-inflammatory cytokines, the improved cell viability of the intestinal model was associated with increased E-cadherin and ZO-1 expression. Interestingly, many lung-on-chip or in vitro approaches like those used in the abovementioned models have used commercially available non-biological scaffolds to build 3D systems like polystyrol membranes [29]. 

### 4.1. 3D Immunocompetent Model System

Although 2D cell culture techniques including organoid cultures have established their importance in cancer cell culture research areas, they exhibit various disadvantages when compared with state-of-the-art, 3D, in vitro systems (Table 1). Among certain limitations, cell–cell and cell–ECM interactions are key aspects when modelling the tumour microenvironment [23]. Loss of cellular phenotype, change in morphology and, hence, functionality, and cell signalling have been observed while the cells are grown in 2D cultures after isolation from their native tissues. This can be explained through the absence of essential stimuli, polarization, and proteins, which are made available through interactions with the extracellular environment. However, monocultural systems prove to be inadequate when modelling various infection pathways, including bacterial infestations [22]. 

Bacteria can essentially surpass into the deeper tissues and can even interact with fibroblasts, which are responsible for modulating and restructuring the ECM [22]. Among many important factors to be considered when designing 3D in vitro tissue models, co-culture condition optimizations, the selection of critical cell types, etc., and the selection and synthesis of the appropriate scaffold structure as an ECM substitute are crucial. Such a scaffold material, which can provide the basis for the extracellular matrix, is required to not only mimic but also to re-enforce cellular and tissue differentiation similar to that in an in vivo environment. SIS-muc is one of the biologically derived scaffold materials that have a wide range of applications in 3D in vitro tissue modelling. SIS-muc is obtained after the decellularization procedure of a porcine jejunum segment using sodium desoxchylate, and the perfusion of an intact vessel network within the intestinal wall is published in [12]. Such a scaffold is measured to provide a thickness of approximately 0.2 ± 0.01 mm. The matrix comprises a complex interlinked fibrous network made of 5% elastin and 92% cross-linked collagen fibres, thereby providing a 3D architecture for cellular attachment similar to that in in vivo conditions [13]. Such an interlinked matrix provides a biomimetic collagen fibre mesh for the fibroblasts to migrate inside the scaffold to be remodelled into a 3D tissue-like structure. SIS-muc has been widely chosen for modelling functional 3D in vitro human upper airway models given its extracellular matrix architecture providing an essential dense layer of cross-linked collagen and an elastin fibre network. Moreover, such a conditioned scaffold can provide better growth conditions for cellular proliferation and tissue differentiation [12,13]. Hence, in our attempt to produce a basic 3D immunocompetent tissue model, we utilised SIS-muc that may provide the added advantage of being a physiologically relevant ECM. In our attempt to produce a 3D immunocompetent tissue model, we selected healthy primary human dermal fibroblasts to produce a simple tissue model, which was then complimented with the addition of second and main role-player monocytes/macrophages in a co-culture. The tissues produced were characterized to ensure cellular confluency using HE staining and were seen to be proliferative given the positive staining against the Ki67 marker in immune histological staining (results not shown). Furthermore, it is known that there is an effect of the hostile environment mediated through the presence of specific phenotypes of macrophages in several bacterial pathogens [16]. Hence, to produce and study an immunocompetent model system, THP-1 monocytes were utilised in vitro to produce two major phenotypes of macrophages, namely M1 and M2 [24]. Furthermore, the differentiation status was characterized using immunofluorescence to ensure the integrity of the microenvironment produced for the establishment of the co-cultures. The heterogenous and plastic behaviour of macrophages makes it difficult to differentiate them in vitro as well as in vivo into a monochrome spectrum that has M1 and M2 macrophages. The heterogeneity in behaviour may be the consequence of a wide range of environmental factors, including cytokines, chemokines, pattern recognition receptors, hormones, and others, differentially regulating the response [30]. Given the stimulative, progressive changes in the microenvironment, which were both in vitro as well as in vivo, the functional response can be dynamic in nature and may change over time [31]. Studies suggest that macrophages can be re-stimulated by the present cytokine milieu and perform a functional shift [32]. In addition to the microenvironment, the incubation times and handling may also affect the polarization of the macrophages. As suggested by D’Andrea et al., macrophages, when polarized towards the M1/M2 phenotypes by exposure to certain cytokines when washed after the intended incubation, may also polarize backward towards their basal state when the corresponding cytokines are removed (given that there are no other infections/inflammation/stimuli present) [33]. Moreover, significant enhancement of TNF-α and IL-12 production was observed when macrophages were treated overnight with IL-4 [33]. A mixture of IL4 and IL13 cytokine stimulation is also connected with the enhanced production of TNF-α and IL12 production after 20 h of incubation, which can explain the heterogenous population that is found in the IF staining since it may induce differentiation in M1-like functioning macrophages [33]. There is no consensus about forced homogeneity in the differentiation/polarization of M1/M2 macrophages derived from THP1 monocyte-like cells [34], which is also reflected in our fluorescence surface characterization of macrophage differentiation. The standardized, established protocol for the differentiation of THP1 monocyte-like cells in vitro into the two distinct macrophage populations of M1 and M2 yielded heterogenous populations, as seen in (Figure 2), since a certain proportion of inflammatory phenotypic macrophages were still found in the population of anti-inflammatory phenotype M2. This reinforces the dynamic and heterogeneous nature of macrophages found in vivo [16,30,35]. Upon establishing the co-culture with pre-differentiated macrophages, the next vital element included was that of the bacterial biofilm. The models were initially exposed to the bacterial biofilm for 3 days to control the bacterial overgrowth and study the impact of the co-culture. Nevertheless, the heterogeneity of the macrophage population and high number of the intended respective M1/M2 population were able to show the impact of the established immunocompetence at the end, which was observed in the mitigation of bacterial growth in the case of M1 (Figure 3).

### 4.2. Immunocompetence

When the tissue sections were monitored for cellular outgrowth, they showed a highly confluent fibroblast monolayer which was made evident by the vimentin-positive fibroblasts in the immunohistology of models without bacterial biofilm exposure. Additionally, the influence of co-cultures of pre-differentiated macrophages was clearly visible on the tissue architecture. Since M1 macrophages are known to be a pro-inflammatory phenotype, they modulate their microenvironment by secreting pro-inflammatory factors [16,36]. Meanwhile, fibroblasts are also known to secret chemo-attractants and other related growth factors, including IL8 and MCP1 [17], while migrating and modulating the ECM matrix [28,37,38]. In contrast, M2 macrophages are considered to be an anti-inflammatory phenotype by promoting wound healing or tissue remodelling [28,36,39]. M0 macrophages are undifferentiated, early versions of macrophages that inherit the ability to differentiate themselves to M1 or M2 macrophages depending on the given environmental stimuli [28,39]. An observation for the tissues in a co-culture with M0 and M2 macrophages appeared similarly densely structured when compared with that with M1 co-cultured tissues, where some cellular migration inside the tissue was also seen. This can be attributed to inflammatory microenvironment conditioning in such a way that more fibroblasts are attracted because of the IL8 and MCP1 expression by the M1 macrophages and resident fibroblasts, thereby obtaining more area for proliferation [38], with MCP-1 starting to promote pro-inflammatory conditioning in return [37]. In contrast, the tissues that were exposed to the biofilm showed dramatically diminished vimentin positively stained fibroblasts. Interestingly, in the case of the co-cultured models with M1 and M0 macrophages, the fibroblasts were still visibly stained as the M0 macrophages have the freedom to polarize towards either the M1 or M2 phenotype upon the given stimulation. Meanwhile, in the case of M2, few to no fibroblasts were seen to be visibly stained with vimentin. Hence, we hypothesized that the macrophages were stimulated by the presence of bacterial stimuli to migrate towards the infected tissue and remodulate the microenvironment, which also depended on the pre-existing condition [16]. 

Furthermore, immunofluorescence staining clarified certain aspects of such a differentiated response due to the presence of the M0, M1, and M2 macrophages in the case of the bacteria-infested tissue models. Additionally, it also proved the heterogeneous behaviour of the macrophages, and it demonstrated that the M2 or M1 populations can also consist of little to few subpopulations of M1 and M2 macrophages or M0 macrophages [35] as there are morphological differences (Figure 2). In addition, they can repolarize towards either side, going from M1 back to M0 or M2 back to the M1 phenotype, and they are dynamic in nature when faced with environmental modulations [16,35]. 

### 4.3. M0 Macrophage Models

It was observed that the macrophages migrated into the tissue scaffold, while some of them retained their M0 phenotype when they were stained with CD68 (Figure 5G). However, certain macrophages differentiated themselves into the M1 inflammatory phenotype, which was stained positive with the marker CD80 (Figure 5E). The presence of a blue smear, as depicted in Figure 5C, showed the presence of bacterial outgrowth lining the apical side of the tissue, which can explain the heavy lysis of the fibroblasts. Eventually, very few healthy fibroblasts (Figure 5H) were visible by vimentin staining; however, most of the cellular material also appeared in the green-smeared structures across the scaffold (Figure 5D,G). This result was supported through visual examination, OD measurements, and a streak test of the supernatants (Figure 3). The highest OD value (Figure 3B) resulted when the supernatant collected after 3 days was analysed, and it showed the highest turbidity visually and generated the highest growth in bacteria when grown on agar plates (Figure 3C). Clinical findings from the serum samples from patients in the study speculated that the Gram-positive bacteria from the blood stream showed exceptionally higher levels of IL4 and IL3 expression [40]. Biofilm models, when co-cultured with M0 macrophages, additionally demonstrated an upregulation of IL4 seen in the multiplex ELISA analysis. We also monitored an upregulation of IL8, but IL6 and MCP1 expressions were significantly lowered. This reinstates the hypothesis that was evaluated based on the histological findings. A significantly higher expression of IL8 from the activated fibroblasts due to the presence of bacteria initiated the recruitment of activated macrophages if present in the culture. While certain M0 macrophages managed to polarize towards the inflammatory phenotype, other M0 macrophages were able to migrate inside the tissue structure, so the culture was seen transiting towards an inflammatory response through the expression of IL6 [16,41]. Outgrowth of the bacterial biofilm was able to be overruled, and the inflammatory response was soon denounced.

### 4.4. M1 Macrophage Models

Meanwhile, when the models exposed to bacteria were co-cultured with polarized M1 macrophages in a pro-inflammatory microenvironment, noticeably higher numbers of healthy fibroblasts stained in vimentin (green) were seen, as shown in Figure 5N,P. In addition to the lysed cellular components observed in the form of a blue smear, certain CD68-positive M0 macrophages were additionally present. M1 macrophages are popularly known to be microbicidal and responsible for post-infectious pathogenesis, but certain findings have suggested that they are more plastic in their behaviour and are capable of undergoing functional changes [16]. Research also suggests that intracellular bacteria are able to modulate the hostile environment formed by M1 macrophages while interfering with their polarization and drastically reducing their microbicidal functions [16]. This could explain the presence of few CD68-positive M0 macrophages within the tissue. Even though the co-culture with the inflammatory M1 macrophages was unsuccessful in completely eliminating the bacterial infestation from the tissue, it did show the lowest OD value comparable to samples with no biofilm, as shown in Figure 3B. Supernatants from M1 tissue models exposed to the biofilm showed no visual turbidity unlike the highly turbid supernatant found in the M0 macrophage models. Hence, M1 macrophage models with the bacterial biofilm generated the lowest outgrowth of bacterial colonies when inoculated on an agar plate, as shown in Figure 3D. Accordingly, healthy fibroblasts stained positive for vimentin were found in histological staining (Figure 4K) and fluorescence staining revealed comparatively higher proportion of healthy fibroblasts along with a certain level of presence of lysed cells in the form of a blue-green smear (Figure 5J,K).

The inflammatory microenvironment created by the M1 macrophages is known to show a characteristic upregulation of certain cytokines, which are mainly IL6, IL12, and IL1β, among the expressions of TNF-α and IFNγ [16]. As expected, in such an environment, exceptionally high levels of MCP1, IL6, and IL8 cytokines were found in the control groups without the bacterial biofilm exposure, attraction, and migration of M1 macrophages, while anti-inflammatory cytokines, specifically IL4 and IL12, remained low, as shown in Figure 6B. Bacterial biofilm exposure seemed to act as a stimulus to aggravate the expression of IL8, which heavily contributes towards pro-inflammatory macrophage activation [38]. Interestingly enough, the inflammatory cytokine IL12p70 did not show a significant difference in its expression when compared with the different infection groups including the Gram-negative and Gram-positive bacterial and fungal infections [40], and it was seen gradually increasing to be significantly higher at day 3 for the M1 co-cultured models with bacteria. IL12p70 is known to play a vital role in participating in inflammatory activation when an infection occurs [40]. Meanwhile, it remained unaffected in the case of the M0 and M2 co-cultures, therefore suggesting that the bacterial infestation was able to initiate an immune response through the cascade of IL8 and IL12p70. On the contrary, no significant expressions were noted for the other pro-inflammatory cytokines, namely TNF-α, IFN-γ, and IL1β, while the expression of IL17 decreased significantly. Studies have investigated the altering effect of certain bacterial species to diverge inflammatory action [17,35,42,43]. For example, in mycobacterial infections, IL6 expression can also alter or hinder IFNγ production, consequently resulting in Q-fever. Certain bacteria are also capable of the M2 reprogramming of macrophages by IL4 or IL10 expression [16]. Similar trends were seen in our investigation as a consequence of the inflammatory environment was not enough to eradicate bacterial infestation fully but up to 80%, as proven through OD measurements as well.

### 4.5. M2 Macrophage Models

In the case of models in a co-culture with an anti-inflammatory phenotype M2 population of macrophages, histological analysis revealed structural loosening along with heavier cell lysis (Figure 4F) as well as diffused and scattered cellular material. Very few healthy fibroblasts were seen to be stained with vimentin in immunohistological findings (Figure 4L). Surprisingly enough, little to no CD163-positive macrophages were found to have successfully migrated inside the tissue, while certain CD80-positive M1 phenotypic macrophages were located encircling the vimentin-stained fibroblast remnants (Figure 5U). Although certain vimentin-stained healthy fibroblasts with intact cell membranes were spotted in the tissue, most of the cells appeared lysed and smeared around the scaffold (Figure 5U,V). Meanwhile, a blue smear was clearly visible depicting the biofilm invasion and cellular lysis through scattered materials stained blue by DAPI (Figure 5X). These things considered, certain CD68-positive M0 macrophages were also spotted. Consequently, the supernatant from the M2 co-cultured models appeared heavily turbid when compared with control M2 co-cultured models without bacteria. Visually, the turbidity level was comparable to that of the M0 co-cultured models with bacteria. Optical density measurements showed a significant increase in the turbidity of M2 with bacteria compared with the corresponding control. Consequently, the M2 models also showed moderate to high bacterial outgrowth when supernatants were inoculated on agar plates at the end of day 3 of the culture (Figure 3E). When the biofilm-exposed tissue was co-cultured in the M2-mediated environment, the presence of IL4 anti-inflammatory cytokines in turn augmented towards the higher production of MCP1 [37], which is evident from the analysis of the supernatant cytokines.

M2 macrophages can exist in different subtypes as follows: M2a/b/c/d. Interestingly, the M2b subtype is popularly known for its protective and pathogenic functions in different diseases. As seen from the macrophage characterization in Figure 2F,I, certain macrophages were also seen expressing CD80 as well as CD163. Similar research findings also suggest that the M2b subtype shows the surface markers of CD80 along with that of CD163 [16]. Certain studies have proved the role of the macrophages of the M2b subtype in promoting tumour development and bacterial, parasite, and fungal infections by lowering the immune or inflammatory responses [39]. Bacteria and viruses are also known to directly promote M2b macrophage polarization with the characteristic low values of IL12 and the expression of IL6 along with TNF-α and IL10. Although these macrophages are highly capable of being phagocytic in nature, they remain unable to eradicate bacterial invasions [39]. Bacterial species like *Staphylococcus aureus* or methicillin-resistant *S. aureus* (MRSA), *E. faecalis*, *C. albicans*, and *K. pneumonia* override the M2b macrophage response, leaving them increasingly susceptible to infectious enhancement [44]. Therefore, our data suggest the hypothesis that the M2 macrophages may have undergone further differentiation into the M2b subtype while still having a heavy expression of MCP1 and IL8, which promoted the induction of the inflammatory response, and the M2b macrophages were able to blunt the immune response. Since they are capable of stopping the repolarization of M0 to M1, this might consequently result in tumour formation.

In addition to the macrophages being responsible for regulating the immune response and counteracting the infection, bacteria and bacterial biofilms are also seen to be strategic manipulators in inhibiting phagocytosis and invading tissues [42,43]. Comparable to our approach, a study by Kaya et al. aimed to examine the impact of biofilms produced by *Pseudomonas aeruginosa* and *Staphylococcus epidermis* through an in vitro 2D model using a culture of PBMCs. These two bacterial species, which are popularly known to cause cystic fibrosis, chronic infections, wound and blood stream infections, among others, are also known to be opportunistic pathogens [42]. After 24 h, within the 2D co-culture of PBMCs with bacterial biofilms, the biofilms mechanically detached from the wells. They observed a difference in the bacterial behaviour and immune response generated when the biofilms were intact or disrupted or when direct planktonic bacteria were exposed. Biofilm formations are facilitated by bacteria for growth, escape microbicidal responses from the microenvironment, and host the immune system by providing a 3D structure. Bacteria including S. epidermis use their biofilm as a shield to hide from immune cells and maintain a low inflammatory environment by inducing lower levels of TNF-α, IL6, IFNγ, and IL12p70 [42]. This can explain the persistence of bacterial outgrowth even in the M1 environment in our models. Contrarily, P. aeruginosa biofilms exhibited higher levels of pro-inflammatory cytokine expression including IL6, TNF-α, and anti-inflammatory IL10 than their planktonic counterparts. This again suggested the use of biofilm-specific components, with the structure being an advantage for bacteria to induce an enhanced cell response [42]. In our approach of co-culturing both these bacterial strains to form a biofilm, we may interpret a counteracting effect to be seen in the case of both the M1 and M2 macrophage co-cultures where inflammatory cytokines, such as TNF-α, IL1β, and IFNγ, remained significantly diminished. The expression of MCP1 and IL6 were not sufficient to completely eradicate bacterial infestations, while such opportunistic bacteria that formed biofilms were successful in strategizing their survival.

In contrast to the published in vitro biofilm 2D model approach explained in [42], to our knowledge, we were able to successfully establish a 3D model system with immunocompetent properties that can for the first time be applicable for biofilm model studies using pre-differentiated inflammatory and anti-inflammatory environments by inclusion of immune cells. Such a 3D model structure can facilitate and help to mimic complex in vivo responses more accurately not only for monocultures but also for advanced, complex, tissue-specific co-cultures in the future.

## 5. Conclusions

In conclusion, our developed immunocompetent human 3D tissue model can be used to monitor tissue changes due to bacterial impact over three days without becoming overwhelmed. Further investigations have to be carried out to increase the time span and to test for transferability to usually tissue-damaging microorganisms. External factors may also be analysed within this context. In our model, antibiotics were added to the medium to limit bacterial growth without killing them as our hypothesis suggests they would have overgrown too quickly without them. Future studies could investigate the effect of different concentrations of antibiotics or the complete absence of them.

## Figures and Tables

**Table 2:** List of antibodies used including the corresponding incubation conditions and dilutions for the immunohistochemical and immunofluorescent analysis.

Antibody	Manufacturer	Incubation Time/ Temperature	Dilution IHC	Dilution IF	Cells Positive
mCD68	Invitrogen, Darmstadt, Germany, 14-0688-82	Overnight, 4 °C	1:500	5 µg/mL	M0
rCD80	Invitrogen, Darmstadt, Germany, PA585913	Overnight, 4 °C	1:500	1:100	M1
rCD163	BIOSUSA, Massachusetts U.S.A. bsm-54015R	Overnight, 4 °C	1:100	1:100	M2
Vimentin	Sigma-Aldrich, Taufkirchen, Germany, V2258	1 h, room temperature	1:400	1:400	Fibroblasts
Anti-mouse IgG FITC	Sigma Aldrich, Taufkirchen, Germany, F0257	1 h, room temperature	--	1:50	--
Anti-rabbit IgG (H+L)	Sigma Aldrich, SAB4600084	1 h, room temperature	--	10 µg/mL	--
DAPI (Fluoromount-G)	Invitrogen, Darmstadt, Germany, E139612	--	--	1 drop/slide	--

## Data Availability

Original data can be provided upon request.

## Supplementary Materials

The following supporting information can be downloaded at: https://www.mdpi.com/article/10.3390/bioengineering11020187/s1, Figure S1: Segmentation of the area corresponding to the tissue.; Figure S2: Segmentation of the IHC positive area within the tissue. Figure S3: Semi_Auto_IHC_Thresholding_GrayLevel.
