# Supplementary material for: Establishment of a Human Immunocompetent 3D Tissue Model to Enable the Long-Term Examination of Biofilm–Tissue Interactions
Source: Bioengineering (Basel). 2024 Feb 15;11(2):187. doi: 10.3390/bioengineering11020187 (PMC10885984; doi:10.3390/bioengineering11020187)
Supplement: Supplementary file 1 [file bioengineering-11-00187-s001.zip › supplementary_bioengineering-2841096.pdf]

bioengineering

MDPI

Article

Establishment of a Human Immunocompetent 3D Tissue Model to enable Long-term Examination of Biofilm-Tissue Interaction

Rasika Murkar[1,†], Charlotte von Heckel[1,†], Heike Walles[1], Theresia Moch[1], Christoph Arens[2], Nikolaos Davaris[2], Andre Weber[3], Werner Zuschratter[3], Sönke Baumann[4], Joerg Reinhardt[5], Sascha Kopp[1,*]

[1] Otto-von-Guericke University Magdeburg, Core Facility Tissue Engineering, Universitätsplatz 2, 39106 Magdeburg, Germany
[2] University Clinic Giessen, Department of Otorhinolaryngology, Head and Neck Surgery, 35392, Giessen, Germany, christoph.arens@hno.med.uni-giessen.de (C.A.)
[3] Leibniz Institute for Neurobiology, 39120 Magdeburg, Germany
[4] Omicron-Laserage® Laserprodukte GmbH Raiffeisenstr. 5e, D-63110 Rodgau, Germany
[5] MedFact Engineering GmbH Hammerstrasse 3, D-79540 Lörrach / Germany
† Authors contributed equally to this manuscript
* Correspondence: sascha.kopp@ovgu.de; +49 3916757488

1. Supplementary

The quantification of the IHC images was done according to the description in methods part. The following images are representatives showing how the process was executed.

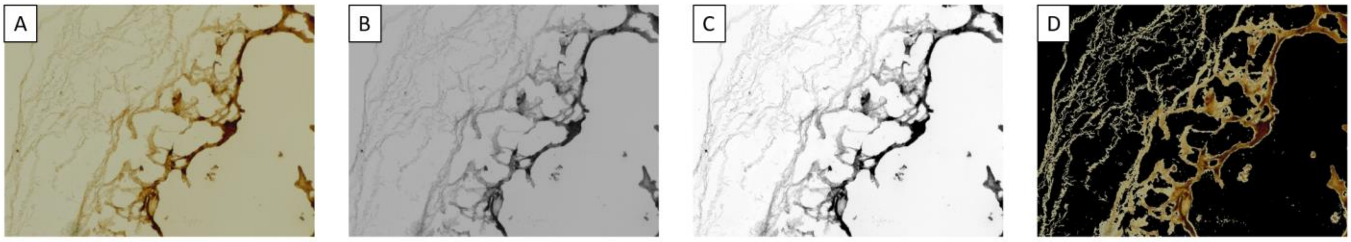

Suppl. Figure S1: Segmentation of the area corresponding to the tissue. (A) Raw RGB image. (B) Grayscale image. (C) Enhanced grayscale image.(D) Segmented tissue form the image.

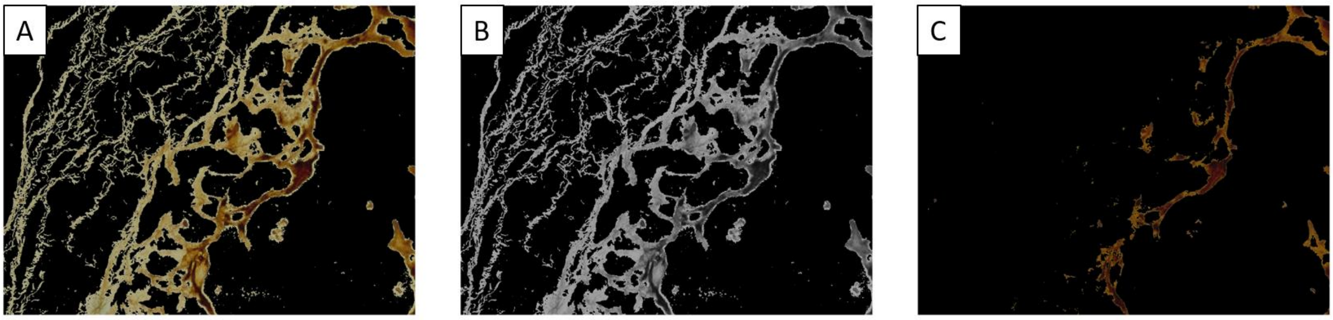

Suppl. Figure S2: Segmentation of the IHC positive area within the tissue. (A) Segmented RGB image. (B) Segmented grayscale image. (C) Segmented IHC positive area within the tissue.
